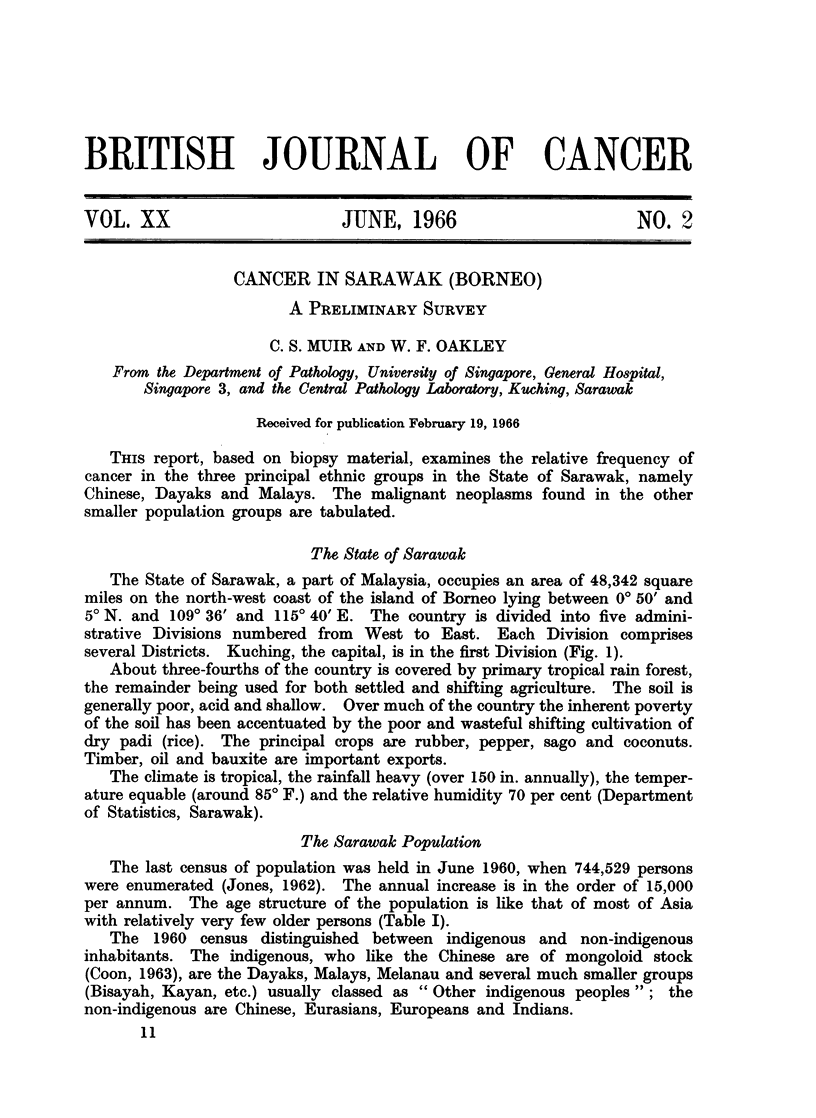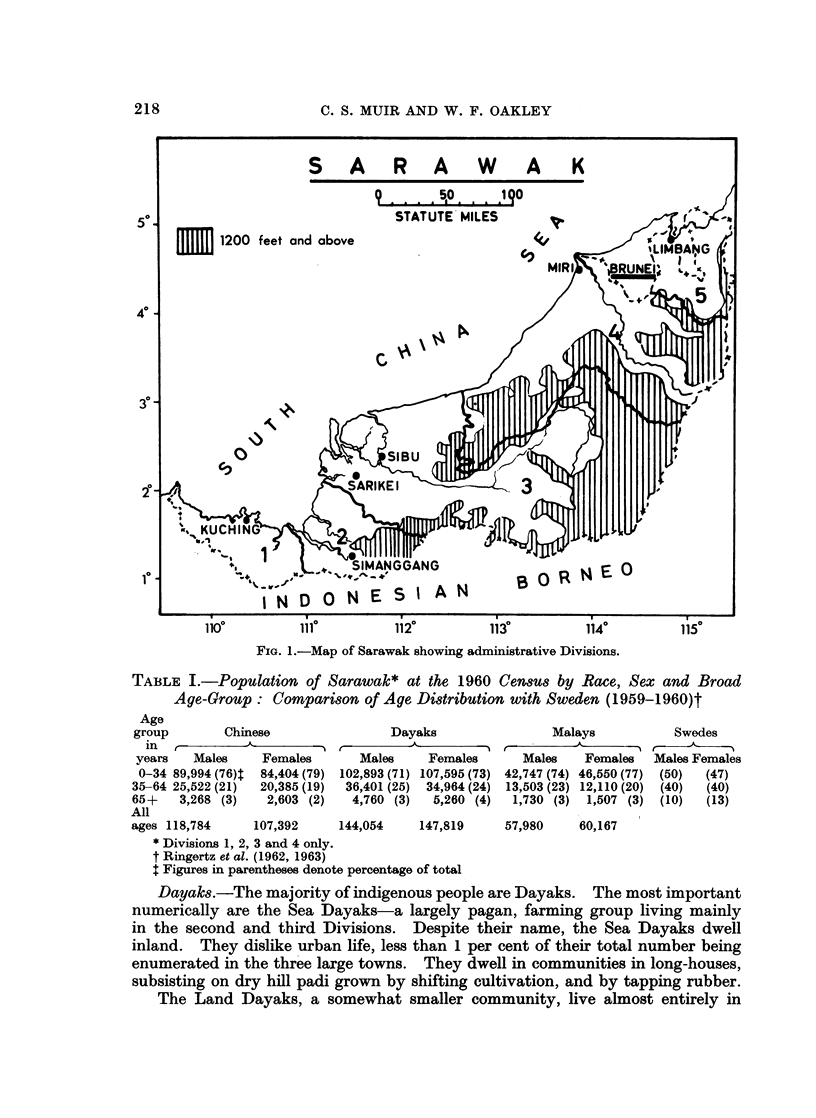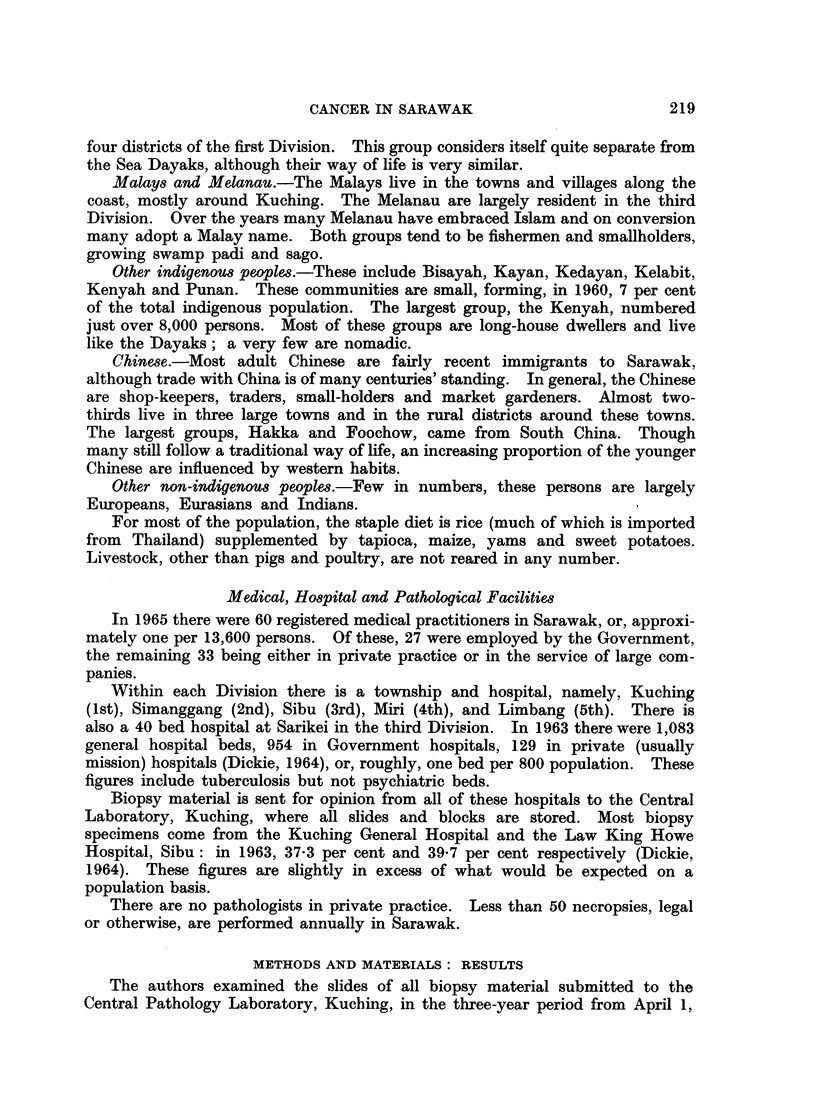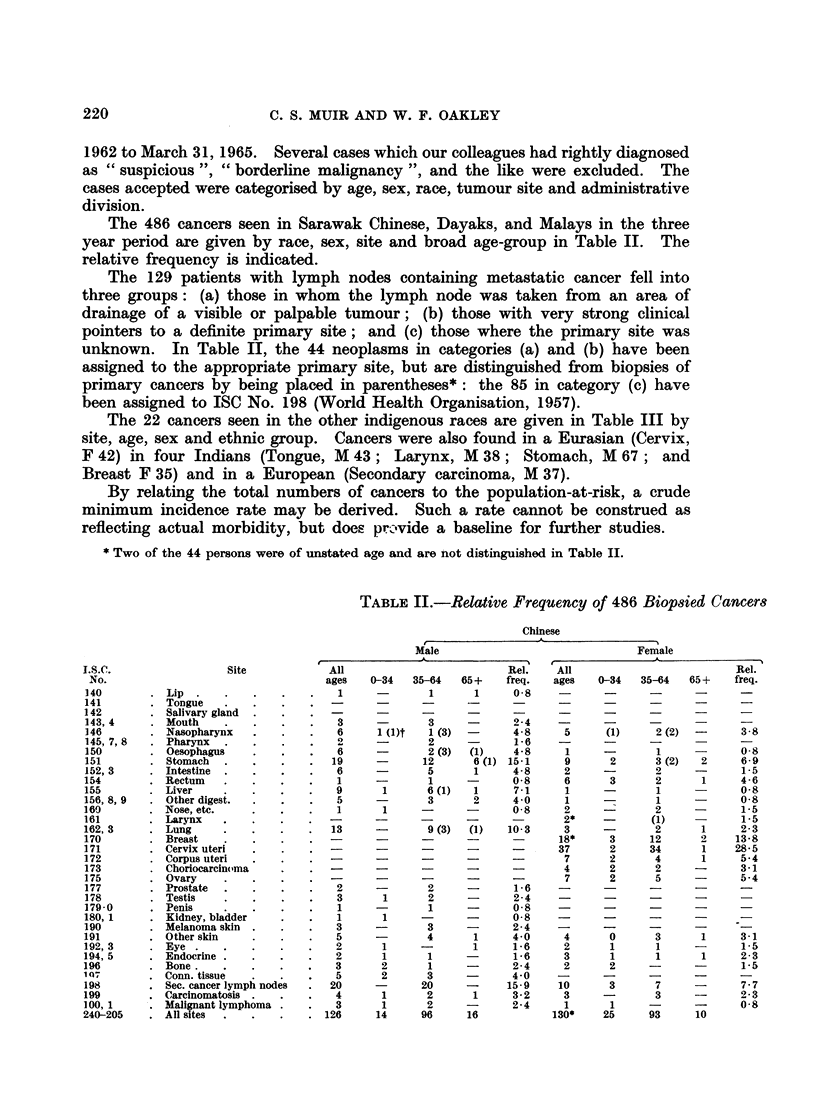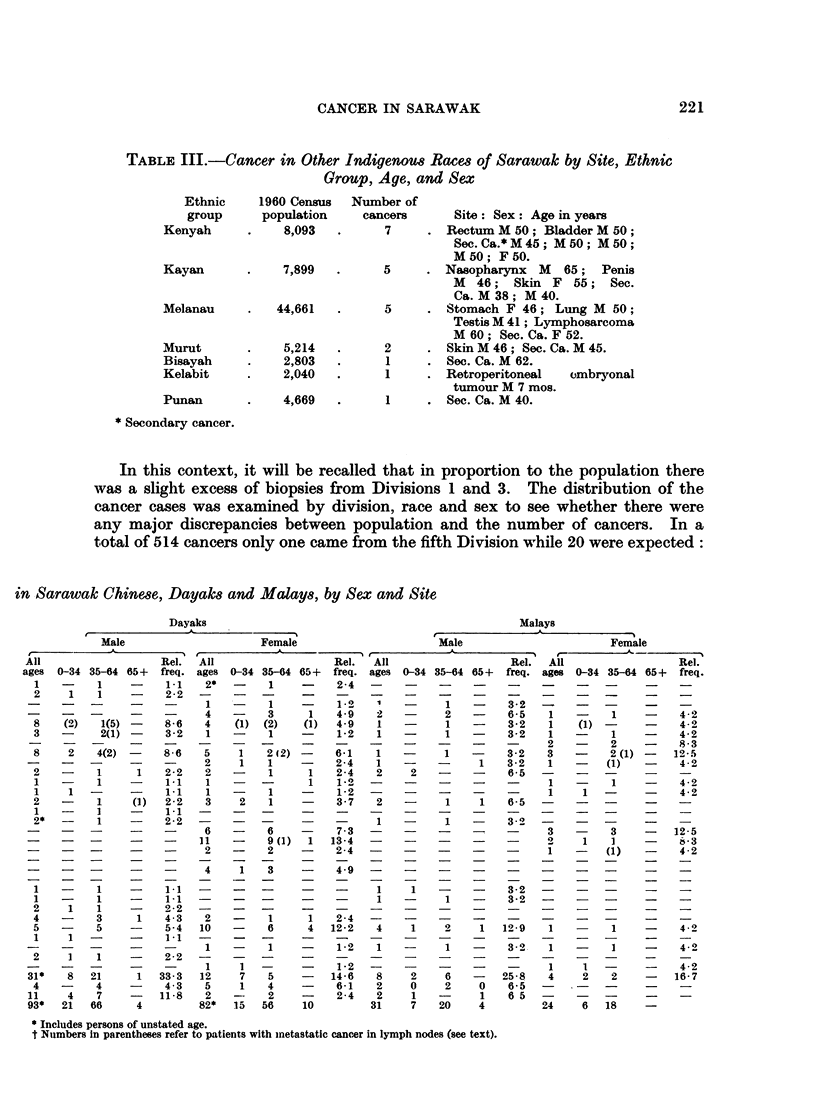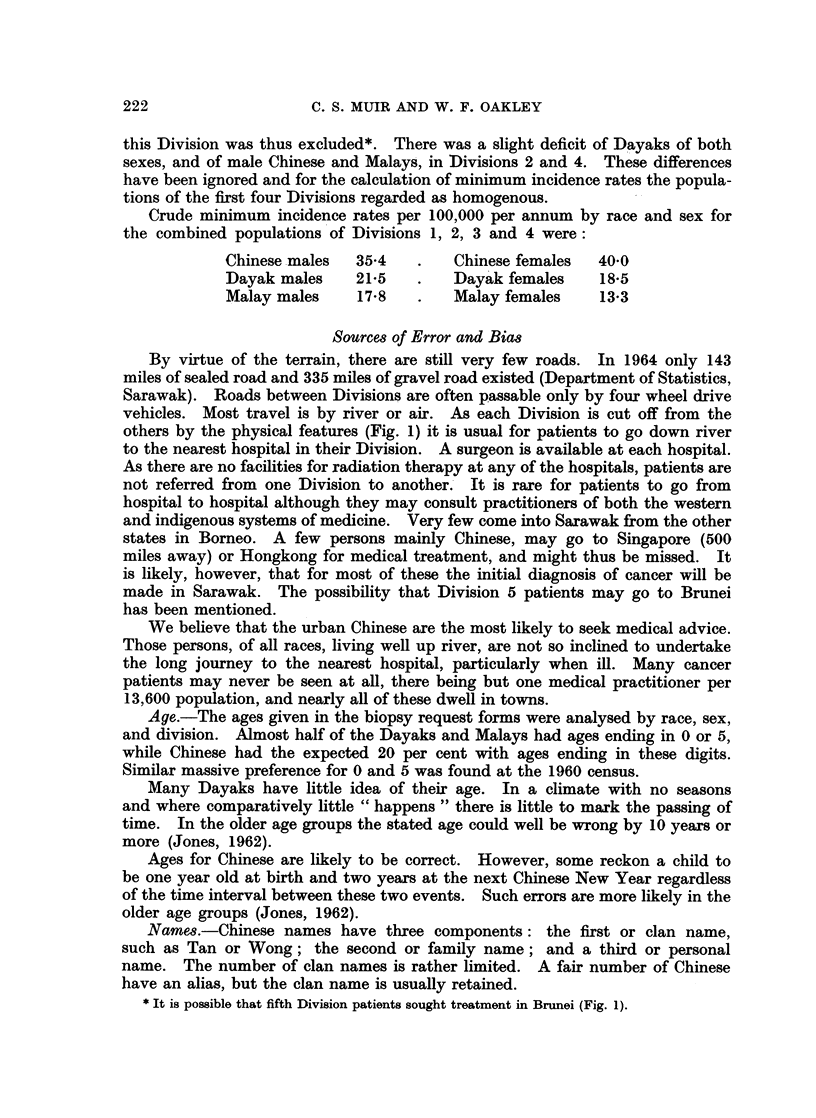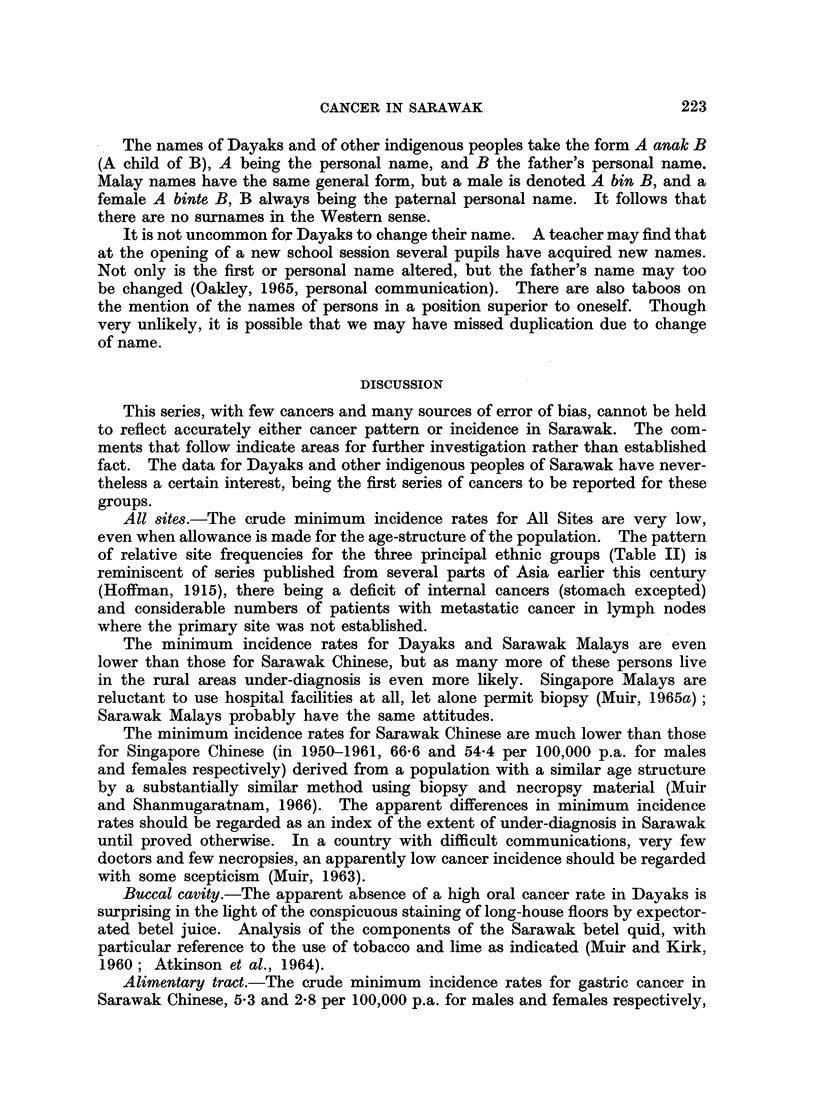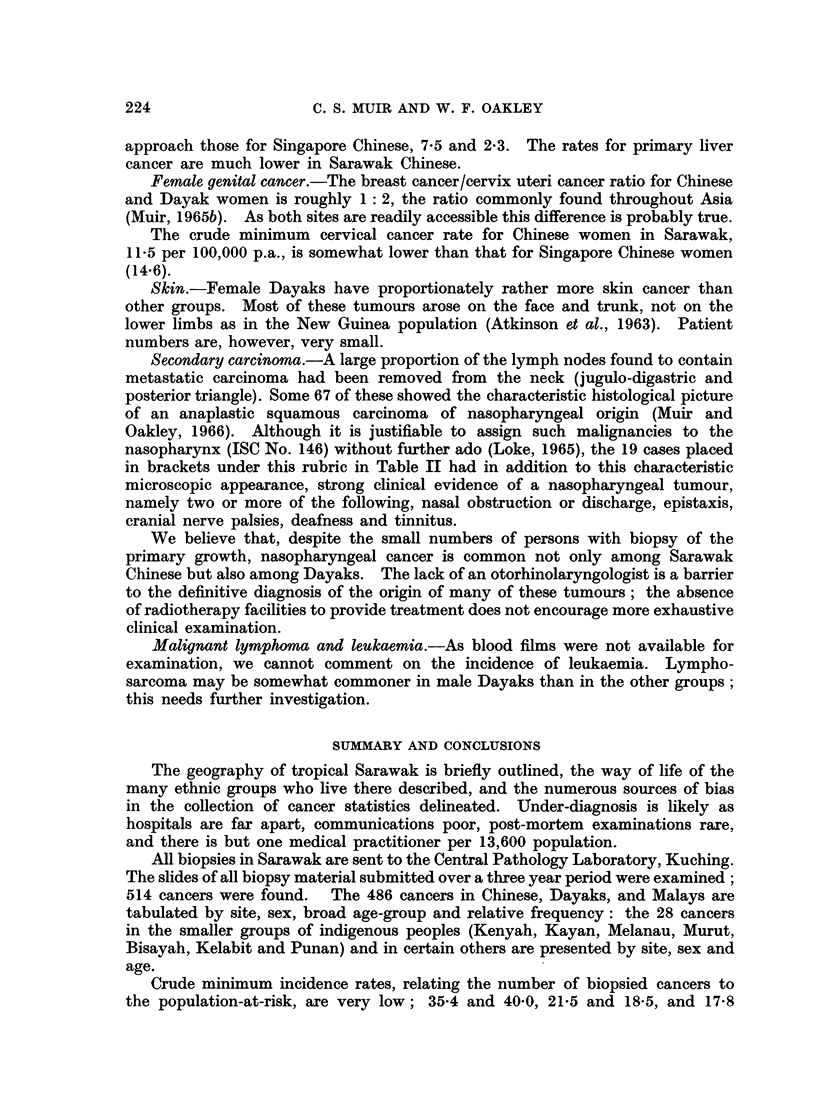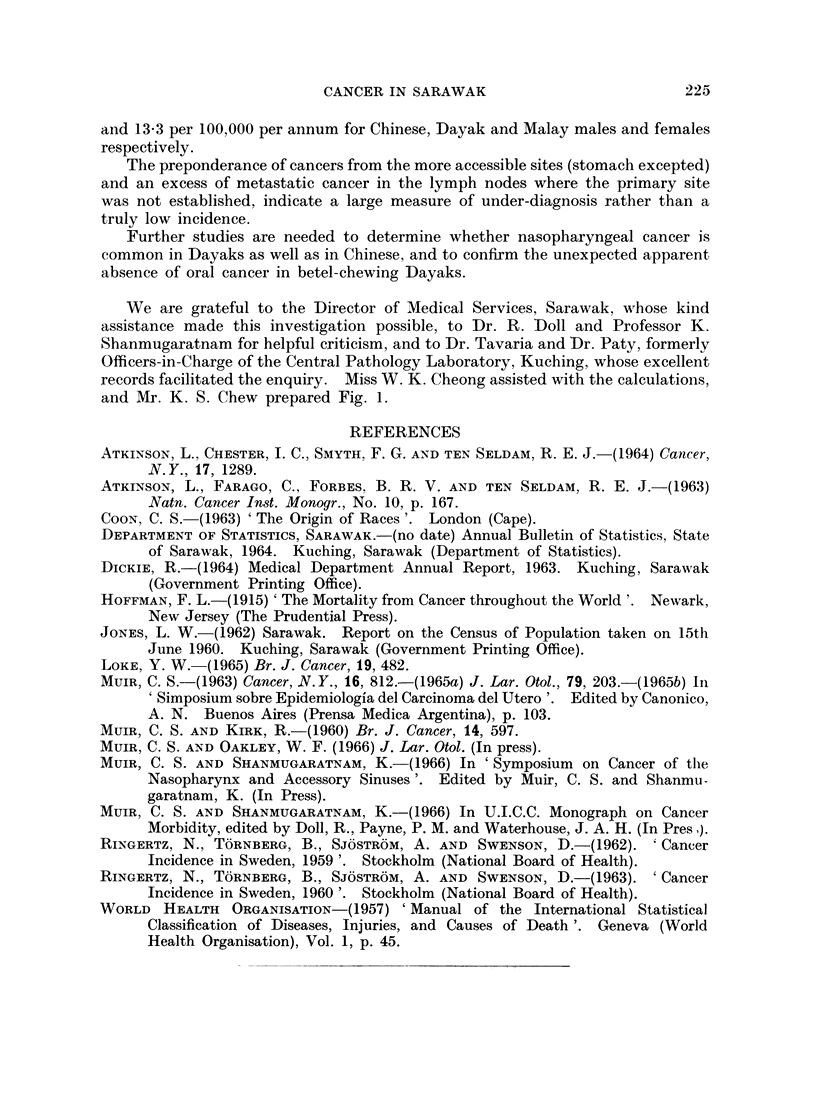# Cancer in Sarawak (Borneo). A preliminary survey.

**DOI:** 10.1038/bjc.1966.26

**Published:** 1966-06

**Authors:** C. S. Muir, W. F. Oakley


### BRITISH JOURNAL OF CANCER

VOL. XX JUNE, 1966 NO. 2

### CANCER IN SARAWAK (BORNEO)

### A PRELIMINARY SURVEY

C. S. MUIR AND W. F. OAKLEY

*From the Department of Pathology, University of Singapore, General Hospital, Singapore 3, and the Central Pathology Laboratory, Kuching, Sarawak*

Received for publication February 19, 1966

THIS report, based on biopsy material, examines the relative frequency of cancer in the three principal ethnic groups in the State of Sarawak, namely Chinese, Dayaks and Malays. The malignant neoplasms found in the other smaller population groups are tabulated.

### *The State of Sarawak*

The State of Sarawak, a part of Malaysia, occupies an area of 48,342 square miles on the north-west coast of the island of Borneo lying between 0° 50′ and 5° N. and 109° 36′ and 115° 40′ E. The country is divided into five administrative Divisions numbered from West to East. Each Division comprises several Districts. Kuching, the capital, is in the first Division (Fig. 1).

About three-fourths of the country is covered by primary tropical rain forest, the remainder being used for both settled and shifting agriculture. The soil is generally poor, acid and shallow. Over much of the country the inherent poverty of the soil has been accentuated by the poor and wasteful shifting cultivation of dry padi (rice). The principal crops are rubber, pepper, sago and coconuts. Timber, oil and bauxite are important exports.

The climate is tropical, the rainfall heavy (over 150 in. annually), the temperature equable (around 85° F.) and the relative humidity 70 per cent (Department of Statistics, Sarawak).

### *The Sarawak Population*

The last census of population was held in June 1960, when 744,529 persons were enumerated (Jones, 1962). The annual increase is in the order of 15,000 per annum. The age structure of the population is like that of most of Asia with relatively very few older persons (Table I).

The 1960 census distinguished between indigenous and non-indigenous inhabitants. The indigenous, who like the Chinese are of mongoloid stock (Coon, 1963), are the Dayaks, Malays, Melanau and several much smaller groups (Bisayah, Kayan, etc.) usually classed as " Other indigenous peoples " ; the non-indigenous are Chinese, Eurasians, Europeans and Indians.

FIG. 1.—Map of Sarawak showing administrative Divisions.

TABLE I.—*Population of Sarawak* at the* 1960 *Census by Race, Sex and Broad Age-Group : Comparison of Age Distribution with Sweden* (1959–1960)†

| Age group in years | Chinese Males | Chinese Females | Dayaks Males | Dayaks Females | Malays Males | Malays Females | Swedes Males | Swedes Females |
|---|---|---|---|---|---|---|---|---|
| 0–34 | 89,994 (76)‡ | 84,404 (79) | 102,893 (71) | 107,595 (73) | 42,747 (74) | 46,550 (77) | (50) | (47) |
| 35–64 | 25,522 (21) | 20,385 (19) | 36,401 (25) | 34,964 (24) | 13,503 (23) | 12,110 (20) | (40) | (40) |
| 65+ | 3,268 (3) | 2,603 (2) | 4,760 (3) | 5,260 (4) | 1,730 (3) | 1,507 (3) | (10) | (13) |
| All ages | 118,784 | 107,392 | 144,054 | 147,819 | 57,980 | 60,167 | | |

* Divisions 1, 2, 3 and 4 only.
† Ringertz *et al.* (1962, 1963)
‡ Figures in parentheses denote percentage of total

*Dayaks.*—The majority of indigenous people are Dayaks. The most important numerically are the Sea Dayaks—a largely pagan, farming group living mainly in the second and third Divisions. Despite their name, the Sea Dayaks dwell inland. They dislike urban life, less than 1 per cent of their total number being enumerated in the three large towns. They dwell in communities in long-houses, subsisting on dry hill padi grown by shifting cultivation, and by tapping rubber.

The Land Dayaks, a somewhat smaller community, live almost entirely in four districts of the first Division. This group considers itself quite separate from the Sea Dayaks, although their way of life is very similar.

*Malays and Melanau.*—The Malays live in the towns and villages along the coast, mostly around Kuching. The Melanau are largely resident in the third Division. Over the years many Melanau have embraced Islam and on conversion many adopt a Malay name. Both groups tend to be fishermen and smallholders, growing swamp padi and sago.

*Other indigenous peoples.*—These include Bisayah, Kayan, Kedayan, Kelabit, Kenyah and Punan. These communities are small, forming, in 1960, 7 per cent of the total indigenous population. The largest group, the Kenyah, numbered just over 8,000 persons. Most of these groups are long-house dwellers and live like the Dayaks ; a very few are nomadic.

*Chinese.*—Most adult Chinese are fairly recent immigrants to Sarawak, although trade with China is of many centuries' standing. In general, the Chinese are shop-keepers, traders, small-holders and market gardeners. Almost two-thirds live in three large towns and in the rural districts around these towns. The largest groups, Hakka and Foochow, came from South China. Though many still follow a traditional way of life, an increasing proportion of the younger Chinese are influenced by western habits.

*Other non-indigenous peoples.*—Few in numbers, these persons are largely Europeans, Eurasians and Indians.

For most of the population, the staple diet is rice (much of which is imported from Thailand) supplemented by tapioca, maize, yams and sweet potatoes. Livestock, other than pigs and poultry, are not reared in any number.

### *Medical, Hospital and Pathological Facilities*

In 1965 there were 60 registered medical practitioners in Sarawak, or, approximately one per 13,600 persons. Of these, 27 were employed by the Government, the remaining 33 being either in private practice or in the service of large companies.

Within each Division there is a township and hospital, namely, Kuching (1st), Simanggang (2nd), Sibu (3rd), Miri (4th), and Limbang (5th). There is also a 40 bed hospital at Sarikei in the third Division. In 1963 there were 1,083 general hospital beds, 954 in Government hospitals, 129 in private (usually mission) hospitals (Dickie, 1964), or, roughly, one bed per 800 population. These figures include tuberculosis but not psychiatric beds.

Biopsy material is sent for opinion from all of these hospitals to the Central Laboratory, Kuching, where all slides and blocks are stored. Most biopsy specimens come from the Kuching General Hospital and the Law King Howe Hospital, Sibu : in 1963, 37·3 per cent and 39·7 per cent respectively (Dickie, 1964). These figures are slightly in excess of what would be expected on a population basis.

There are no pathologists in private practice. Less than 50 necropsies, legal or otherwise, are performed annually in Sarawak.

### METHODS AND MATERIALS : RESULTS

The authors examined the slides of all biopsy material submitted to the Central Pathology Laboratory, Kuching, in the three-year period from April 1,

1962 to March 31, 1965. Several cases which our colleagues had rightly diagnosed as “ suspicious ”, “ borderline malignancy ”, and the like were excluded. The cases accepted were categorised by age, sex, race, tumour site and administrative division.

The 486 cancers seen in Sarawak Chinese, Dayaks, and Malays in the three year period are given by race, sex, site and broad age-group in Table II. The relative frequency is indicated.

The 129 patients with lymph nodes containing metastatic cancer fell into three groups : (a) those in whom the lymph node was taken from an area of drainage of a visible or palpable tumour ; (b) those with very strong clinical pointers to a definite primary site ; and (c) those where the primary site was unknown. In Table II, the 44 neoplasms in categories (a) and (b) have been assigned to the appropriate primary site, but are distinguished from biopsies of primary cancers by being placed in parentheses* : the 85 in category (c) have been assigned to ISC No. 198 (World Health Organisation, 1957).

The 22 cancers seen in the other indigenous races are given in Table III by site, age, sex and ethnic group. Cancers were also found in a Eurasian (Cervix, F 42) in four Indians (Tongue, M 43 ; Larynx, M 38 ; Stomach, M 67 ; and Breast F 35) and in a European (Secondary carcinoma, M 37).

By relating the total numbers of cancers to the population-at-risk, a crude minimum incidence rate may be derived. Such a rate cannot be construed as reflecting actual morbidity, but does provide a baseline for further studies.

* Two of the 44 persons were of unstated age and are not distinguished in Table II.

TABLE II.—*Relative Frequency of* 486 *Biopsied Cancers*

| | | Chinese | | | | | | | | | |
|---|---|---|---|---|---|---|---|---|---|---|---|
| | | Male | | | | | Female | | | | |
| I.S.C. No. | Site | All ages | 0–34 | 35–64 | 65+ | Rel. freq. | All ages | 0–34 | 35–64 | 65+ | Rel. freq. |
| 140 | Lip | 1 | — | 1 | 1 | 0·8 | — | — | — | — | — |
| 141 | Tongue | — | — | — | — | — | — | — | — | — | — |
| 142 | Salivary gland | — | — | — | — | — | — | — | — | — | — |
| 143, 4 | Mouth | 3 | — | 3 | — | 2·4 | — | — | — | — | — |
| 146 | Nasopharynx | 6 | 1 (1)† | 1 (3) | — | 4·8 | 5 | (1) | 2 (2) | — | 3·8 |
| 145, 7, 8 | Pharynx | 2 | — | 2 | — | 1·6 | — | — | — | — | — |
| 150 | Oesophagus | 6 | — | 2 (3) | (1) | 4·8 | 1 | — | 1 | — | 0·8 |
| 151 | Stomach | 19 | — | 12 | 6 (1) | 15·1 | 9 | 2 | 3 (2) | 2 | 6·9 |
| 152, 3 | Intestine | 6 | — | 5 | 1 | 4·8 | 2 | — | 2 | — | 1·5 |
| 154 | Rectum | 1 | — | 1 | — | 0·8 | 6 | 3 | 2 | 1 | 4·6 |
| 155 | Liver | 9 | 1 | 6 (1) | 1 | 7·1 | 1 | — | 1 | — | 0·8 |
| 156, 8, 9 | Other digest. | 5 | — | 3 | 2 | 4·0 | 1 | — | 1 | — | 0·8 |
| 160 | Nose, etc. | 1 | 1 | — | — | 0·8 | 2 | — | 2 | — | 1·5 |
| 161 | Larynx | — | — | — | — | — | 2* | — | (1) | — | 1·5 |
| 162, 3 | Lung | 13 | — | 9 (3) | (1) | 10·3 | 3 | — | 2 | 1 | 2·3 |
| 170 | Breast | — | — | — | — | — | 18* | 3 | 12 | 2 | 13·8 |
| 171 | Cervix uteri | — | — | — | — | — | 37 | 2 | 34 | 1 | 28·5 |
| 172 | Corpus uteri | — | — | — | — | — | 7 | 2 | 4 | 1 | 5·4 |
| 173 | Choriocarcinoma | — | — | — | — | — | 4 | 2 | 2 | — | 3·1 |
| 175 | Ovary | — | — | — | — | — | 7 | 2 | 5 | — | 5·4 |
| 177 | Prostate | 2 | — | 2 | — | 1·6 | — | — | — | — | — |
| 178 | Testis | 3 | 1 | 2 | — | 2·4 | — | — | — | — | — |
| 179·0 | Penis | 1 | — | 1 | — | 0·8 | — | — | — | — | — |
| 180, 1 | Kidney, bladder | 1 | 1 | — | — | 0·8 | — | — | — | — | — |
| 190 | Melanoma skin | 3 | — | 3 | — | 2·4 | — | — | — | — | — |
| 191 | Other skin | 5 | — | 4 | 1 | 4·0 | 4 | 0 | 3 | 1 | 3·1 |
| 192, 3 | Eye | 2 | 1 | — | 1 | 1·6 | 2 | 1 | 1 | — | 1·5 |
| 194, 5 | Endocrine | 2 | 1 | 1 | — | 1·6 | 3 | 1 | 1 | 1 | 2·3 |
| 196 | Bone | 3 | 2 | 1 | — | 2·4 | 2 | 2 | — | — | 1·5 |
| 1[illegible]7 | Conn. tissue | 5 | 2 | 3 | — | 4·0 | — | — | — | — | — |
| 198 | Sec. cancer lymph nodes | 20 | — | 20 | — | 15·9 | 10 | 3 | 7 | — | 7·7 |
| 199 | Carcinomatosis | 4 | 1 | 2 | 1 | 3·2 | 3 | — | 3 | — | 2·3 |
| 100, 1 | Malignant lymphoma | 3 | 1 | 2 | — | 2·4 | 1 | 1 | — | — | 0·8 |
| 240–205 | All sites | 126 | 14 | 96 | 16 | | 130* | 25 | 93 | 10 | |

TABLE III.—*Cancer in Other Indigenous Races of Sarawak by Site, Ethnic Group, Age, and Sex*

| Ethnic group | 1960 Census population | Number of cancers | Site : Sex : Age in years |
|---|---|---|---|
| Kenyah | 8,093 | 7 | Rectum M 50 ; Bladder M 50 ; Sec. Ca.* M 45 ; M 50 ; M 50 ; M 50 ; F 50. |
| Kayan | 7,899 | 5 | Nasopharynx M 65 ; Penis M 46 ; Skin F 55 ; Sec. Ca. M 38 ; M 40. |
| Melanau | 44,661 | 5 | Stomach F 46 ; Lung M 50 ; Testis M 41 ; Lymphosarcoma M 60 ; Sec. Ca. F 52. |
| Murut | 5,214 | 2 | Skin M 46 ; Sec. Ca. M 45. |
| Bisayah | 2,803 | 1 | Sec. Ca. M 62. |
| Kelabit | 2,040 | 1 | Retroperitoneal embryonal tumour M 7 mos. |
| Punan | 4,669 | 1 | Sec. Ca. M 40. |

* Secondary cancer.

In this context, it will be recalled that in proportion to the population there was a slight excess of biopsies from Divisions 1 and 3. The distribution of the cancer cases was examined by division, race and sex to see whether there were any major discrepancies between population and the number of cancers. In a total of 514 cancers only one came from the fifth Division while 20 were expected :

*in Sarawak Chinese, Dayaks and Malays, by Sex and Site*

| Dayaks | | | | | | | | | | Malays | | | | | | | | | |
|---|---|---|---|---|---|---|---|---|---|---|---|---|---|---|---|---|---|---|---|
| Male | | | | | Female | | | | | Male | | | | | Female | | | | |
| All ages | 0–34 | 35–64 | 65+ | Rel. freq. | All ages | 0–34 | 35–64 | 65+ | Rel. freq. | All ages | 0–34 | 35–64 | 65+ | Rel. freq. | All ages | 0–34 | 35–64 | 65+ | Rel. freq. |
| 1 | — | 1 | — | 1·1 | 2* | — | 1 | — | 2·4 | — | — | — | — | — | — | — | — | — | — |
| 2 | 1 | 1 | — | 2·2 | — | — | — | — | — | — | — | — | — | — | — | — | — | — | — |
| — | — | — | — | — | 1 | — | 1 | — | 1·2 | 1 | — | 1 | — | 3·2 | — | — | — | — | — |
| — | — | — | — | — | 4 | — | 3 | 1 | 4·9 | 2 | — | 2 | — | 6·5 | 1 | — | 1 | — | 4·2 |
| 8 | (2) | 1(5) | — | 8·6 | 4 | (1) | (2) | (1) | 4·9 | 1 | — | 1 | — | 3·2 | 1 | (1) | — | — | 4·2 |
| 3 | — | 2(1) | — | 3·2 | 1 | — | 1 | — | 1·2 | 1 | — | 1 | — | 3·2 | 1 | — | 1 | — | 4·2 |
| — | — | — | — | — | — | — | — | — | — | — | — | — | — | — | 2 | — | 2 | — | 8·3 |
| 8 | 2 | 4(2) | — | 8·6 | 5 | 1 | 2 (2) | — | 6·1 | 1 | — | 1 | — | 3·2 | 3 | — | 2 (1) | — | 12·5 |
| — | — | — | — | — | 2 | 1 | 1 | — | 2·4 | 1 | — | — | 1 | 3·2 | 1 | — | (1) | — | 4·2 |
| 2 | — | 1 | 1 | 2·2 | 2 | — | 1 | 1 | 2·4 | 2 | 2 | — | — | 6·5 | — | — | — | — | — |
| 1 | — | 1 | — | 1·1 | 1 | — | — | 1 | 1·2 | — | — | — | — | — | 1 | — | 1 | — | 4·2 |
| 1 | 1 | — | — | 1·1 | 1 | — | 1 | — | 1·2 | — | — | — | — | — | 1 | 1 | — | — | 4·2 |
| 2 | — | 1 | (1) | 2·2 | 3 | 2 | 1 | — | 3·7 | 2 | — | 1 | 1 | 6·5 | — | — | — | — | — |
| 1 | — | 1 | — | 1·1 | — | — | — | — | — | — | — | — | — | — | — | — | — | — | — |
| 2* | — | 1 | — | 2·2 | — | — | — | — | — | 1 | — | 1 | — | 3·2 | — | — | — | — | — |
| — | — | — | — | — | 6 | — | 6 | — | 7·3 | — | — | — | — | — | 3 | — | 3 | — | 12·5 |
| — | — | — | — | — | 11 | — | 9 (1) | 1 | 13·4 | — | — | — | — | — | 2 | 1 | 1 | — | 8·3 |
| — | — | — | — | — | 2 | — | 2 | — | 2·4 | — | — | — | — | — | 1 | — | (1) | — | 4·2 |
| — | — | — | — | — | — | — | — | — | — | — | — | — | — | — | — | — | — | — | — |
| — | — | — | — | — | 4 | 1 | 3 | — | 4·9 | — | — | — | — | — | — | — | — | — | — |
| — | — | — | — | — | — | — | — | — | — | — | — | — | — | — | — | — | — | — | — |
| 1 | — | 1 | — | 1·1 | — | — | — | — | — | 1 | 1 | — | — | 3·2 | — | — | — | — | — |
| 1 | — | 1 | — | 1·1 | — | — | — | — | — | 1 | — | 1 | — | 3·2 | — | — | — | — | — |
| 2 | 1 | 1 | — | 2·2 | — | — | — | — | — | — | — | — | — | — | — | — | — | — | — |
| 4 | — | 3 | 1 | 4·3 | 2 | — | 1 | 1 | 2·4 | — | — | — | — | — | — | — | — | — | — |
| 5 | — | 5 | — | 5·4 | 10 | — | 6 | 4 | 12·2 | 4 | 1 | 2 | 1 | 12·9 | 1 | — | 1 | — | 4·2 |
| 1 | 1 | — | — | 1·1 | — | — | — | — | — | — | — | — | — | — | — | — | — | — | — |
| — | — | — | — | — | 1 | — | 1 | — | 1·2 | 1 | — | 1 | — | 3·2 | 1 | — | 1 | — | 4·2 |
| 2 | 1 | 1 | — | 2·2 | — | — | — | — | — | — | — | — | — | — | — | — | — | — | — |
| — | — | — | — | — | 1 | 1 | — | — | 1·2 | — | — | — | — | — | 1 | 1 | — | — | 4·2 |
| 31* | 8 | 21 | 1 | 33·3 | 12 | 7 | 5 | — | 14·6 | 8 | 2 | 6 | — | 25·8 | 4 | 2 | 2 | — | 16·7 |
| 4 | — | 4 | — | 4·3 | 5 | 1 | 4 | — | 6·1 | 2 | 0 | 2 | 0 | 6·5 | — | — | — | — | — |
| 11 | 4 | 7 | — | 11·8 | 2 | — | 2 | — | 2·4 | 2 | 1 | — | 1 | 6 5 | — | — | — | — | — |
| 93* | 21 | 66 | 4 | | 82* | 15 | 56 | 10 | | 31 | 7 | 20 | 4 | | 24 | 6 | 18 | — | |

* Includes persons of unstated age.
† Numbers in parentheses refer to patients with metastatic cancer in lymph nodes (see text).

this Division was thus excluded*. There was a slight deficit of Dayaks of both sexes, and of male Chinese and Malays, in Divisions 2 and 4. These differences have been ignored and for the calculation of minimum incidence rates the populations of the first four Divisions regarded as homogenous.

Crude minimum incidence rates per 100,000 per annum by race and sex for the combined populations of Divisions 1, 2, 3 and 4 were:

| | | | | |
|---|---|---|---|---|
| Chinese males | 35·4 | . | Chinese females | 40·0 |
| Dayak males | 21·5 | . | Dayak females | 18·5 |
| Malay males | 17·8 | . | Malay females | 13·3 |

*Sources of Error and Bias*

By virtue of the terrain, there are still very few roads. In 1964 only 143 miles of sealed road and 335 miles of gravel road existed (Department of Statistics, Sarawak). Roads between Divisions are often passable only by four wheel drive vehicles. Most travel is by river or air. As each Division is cut off from the others by the physical features (Fig. 1) it is usual for patients to go down river to the nearest hospital in their Division. A surgeon is available at each hospital. As there are no facilities for radiation therapy at any of the hospitals, patients are not referred from one Division to another. It is rare for patients to go from hospital to hospital although they may consult practitioners of both the western and indigenous systems of medicine. Very few come into Sarawak from the other states in Borneo. A few persons mainly Chinese, may go to Singapore (500 miles away) or Hongkong for medical treatment, and might thus be missed. It is likely, however, that for most of these the initial diagnosis of cancer will be made in Sarawak. The possibility that Division 5 patients may go to Brunei has been mentioned.

We believe that the urban Chinese are the most likely to seek medical advice. Those persons, of all races, living well up river, are not so inclined to undertake the long journey to the nearest hospital, particularly when ill. Many cancer patients may never be seen at all, there being but one medical practitioner per 13,600 population, and nearly all of these dwell in towns.

*Age.*—The ages given in the biopsy request forms were analysed by race, sex, and division. Almost half of the Dayaks and Malays had ages ending in 0 or 5, while Chinese had the expected 20 per cent with ages ending in these digits. Similar massive preference for 0 and 5 was found at the 1960 census.

Many Dayaks have little idea of their age. In a climate with no seasons and where comparatively little " happens " there is little to mark the passing of time. In the older age groups the stated age could well be wrong by 10 years or more (Jones, 1962).

Ages for Chinese are likely to be correct. However, some reckon a child to be one year old at birth and two years at the next Chinese New Year regardless of the time interval between these two events. Such errors are more likely in the older age groups (Jones, 1962).

*Names.*—Chinese names have three components: the first or clan name, such as Tan or Wong; the second or family name; and a third or personal name. The number of clan names is rather limited. A fair number of Chinese have an alias, but the clan name is usually retained.

* It is possible that fifth Division patients sought treatment in Brunei (Fig. 1).

The names of Dayaks and of other indigenous peoples take the form *A anak B* (A child of B), *A* being the personal name, and *B* the father's personal name. Malay names have the same general form, but a male is denoted *A bin B*, and a female *A binte B*, B always being the paternal personal name. It follows that there are no surnames in the Western sense.

It is not uncommon for Dayaks to change their name. A teacher may find that at the opening of a new school session several pupils have acquired new names. Not only is the first or personal name altered, but the father's name may too be changed (Oakley, 1965, personal communication). There are also taboos on the mention of the names of persons in a position superior to oneself. Though very unlikely, it is possible that we may have missed duplication due to change of name.

### DISCUSSION

This series, with few cancers and many sources of error of bias, cannot be held to reflect accurately either cancer pattern or incidence in Sarawak. The comments that follow indicate areas for further investigation rather than established fact. The data for Dayaks and other indigenous peoples of Sarawak have nevertheless a certain interest, being the first series of cancers to be reported for these groups.

*All sites.*—The crude minimum incidence rates for All Sites are very low, even when allowance is made for the age-structure of the population. The pattern of relative site frequencies for the three principal ethnic groups (Table II) is reminiscent of series published from several parts of Asia earlier this century (Hoffman, 1915), there being a deficit of internal cancers (stomach excepted) and considerable numbers of patients with metastatic cancer in lymph nodes where the primary site was not established.

The minimum incidence rates for Dayaks and Sarawak Malays are even lower than those for Sarawak Chinese, but as many more of these persons live in the rural areas under-diagnosis is even more likely. Singapore Malays are reluctant to use hospital facilities at all, let alone permit biopsy (Muir, 1965*a*); Sarawak Malays probably have the same attitudes.

The minimum incidence rates for Sarawak Chinese are much lower than those for Singapore Chinese (in 1950–1961, 66·6 and 54·4 per 100,000 p.a. for males and females respectively) derived from a population with a similar age structure by a substantially similar method using biopsy and necropsy material (Muir and Shanmugaratnam, 1966). The apparent differences in minimum incidence rates should be regarded as an index of the extent of under-diagnosis in Sarawak until proved otherwise. In a country with difficult communications, very few doctors and few necropsies, an apparently low cancer incidence should be regarded with some scepticism (Muir, 1963).

*Buccal cavity.*—The apparent absence of a high oral cancer rate in Dayaks is surprising in the light of the conspicuous staining of long-house floors by expectorated betel juice. Analysis of the components of the Sarawak betel quid, with particular reference to the use of tobacco and lime as indicated (Muir and Kirk, 1960; Atkinson *et al.*, 1964).

*Alimentary tract.*—The crude minimum incidence rates for gastric cancer in Sarawak Chinese, 5·3 and 2·8 per 100,000 p.a. for males and females respectively, approach those for Singapore Chinese, 7·5 and 2·3. The rates for primary liver cancer are much lower in Sarawak Chinese.

*Female genital cancer.*—The breast cancer/cervix uteri cancer ratio for Chinese and Dayak women is roughly 1 : 2, the ratio commonly found throughout Asia (Muir, 1965*b*). As both sites are readily accessible this difference is probably true.

The crude minimum cervical cancer rate for Chinese women in Sarawak, 11·5 per 100,000 p.a., is somewhat lower than that for Singapore Chinese women (14·6).

*Skin.*—Female Dayaks have proportionately rather more skin cancer than other groups. Most of these tumours arose on the face and trunk, not on the lower limbs as in the New Guinea population (Atkinson *et al.*, 1963). Patient numbers are, however, very small.

*Secondary carcinoma.*—A large proportion of the lymph nodes found to contain metastatic carcinoma had been removed from the neck (jugulo-digastric and posterior triangle). Some 67 of these showed the characteristic histological picture of an anaplastic squamous carcinoma of nasopharyngeal origin (Muir and Oakley, 1966). Although it is justifiable to assign such malignancies to the nasopharynx (ISC No. 146) without further ado (Loke, 1965), the 19 cases placed in brackets under this rubric in Table II had in addition to this characteristic microscopic appearance, strong clinical evidence of a nasopharyngeal tumour, namely two or more of the following, nasal obstruction or discharge, epistaxis, cranial nerve palsies, deafness and tinnitus.

We believe that, despite the small numbers of persons with biopsy of the primary growth, nasopharyngeal cancer is common not only among Sarawak Chinese but also among Dayaks. The lack of an otorhinolaryngologist is a barrier to the definitive diagnosis of the origin of many of these tumours ; the absence of radiotherapy facilities to provide treatment does not encourage more exhaustive clinical examination.

*Malignant lymphoma and leukaemia.*—As blood films were not available for examination, we cannot comment on the incidence of leukaemia. Lymphosarcoma may be somewhat commoner in male Dayaks than in the other groups ; this needs further investigation.

### SUMMARY AND CONCLUSIONS

The geography of tropical Sarawak is briefly outlined, the way of life of the many ethnic groups who live there described, and the numerous sources of bias in the collection of cancer statistics delineated. Under-diagnosis is likely as hospitals are far apart, communications poor, post-mortem examinations rare, and there is but one medical practitioner per 13,600 population.

All biopsies in Sarawak are sent to the Central Pathology Laboratory, Kuching. The slides of all biopsy material submitted over a three year period were examined ; 514 cancers were found. The 486 cancers in Chinese, Dayaks, and Malays are tabulated by site, sex, broad age-group and relative frequency : the 28 cancers in the smaller groups of indigenous peoples (Kenyah, Kayan, Melanau, Murut, Bisayah, Kelabit and Punan) and in certain others are presented by site, sex and age.

Crude minimum incidence rates, relating the number of biopsied cancers to the population-at-risk, are very low ; 35·4 and 40·0, 21·5 and 18·5, and 17·8

and 13·3 per 100,000 per annum for Chinese, Dayak and Malay males and females respectively.

The preponderance of cancers from the more accessible sites (stomach excepted) and an excess of metastatic cancer in the lymph nodes where the primary site was not established, indicate a large measure of under-diagnosis rather than a truly low incidence.

Further studies are needed to determine whether nasopharyngeal cancer is common in Dayaks as well as in Chinese, and to confirm the unexpected apparent absence of oral cancer in betel-chewing Dayaks.

We are grateful to the Director of Medical Services, Sarawak, whose kind assistance made this investigation possible, to Dr. R. Doll and Professor K. Shanmugaratnam for helpful criticism, and to Dr. Tavaria and Dr. Paty, formerly Officers-in-Charge of the Central Pathology Laboratory, Kuching, whose excellent records facilitated the enquiry. Miss W. K. Cheong assisted with the calculations, and Mr. K. S. Chew prepared Fig. 1.

### REFERENCES

Atkinson, L., Chester, I. C., Smyth, F. G. and ten Seldam, R. E. J.—(1964) *Cancer, N.Y.*, **17**, 1289.

Atkinson, L., Farago, C., Forbes, B. R. V. and ten Seldam, R. E. J.—(1963) *Natn. Cancer Inst. Monogr.*, No. 10, p. 167.

Coon, C. S.—(1963) ' The Origin of Races '. London (Cape).

Department of Statistics, Sarawak.—(no date) Annual Bulletin of Statistics, State of Sarawak, 1964. Kuching, Sarawak (Department of Statistics).

Dickie, R.—(1964) Medical Department Annual Report, 1963. Kuching, Sarawak (Government Printing Office).

Hoffman, F. L.—(1915) ' The Mortality from Cancer throughout the World '. Newark, New Jersey (The Prudential Press).

Jones, L. W.—(1962) Sarawak. Report on the Census of Population taken on 15th June 1960. Kuching, Sarawak (Government Printing Office).

Loke, Y. W.—(1965) *Br. J. Cancer*, **19**, 482.

Muir, C. S.—(1963) *Cancer, N.Y.*, **16**, 812.—(1965*a*) *J. Lar. Otol.*, **79**, 203.—(1965*b*) In ' Simposium sobre Epidemiología del Carcinoma del Utero '. Edited by Canonico, A. N. Buenos Aires (Prensa Medica Argentina), p. 103.

Muir, C. S. and Kirk, R.—(1960) *Br. J. Cancer*, **14**, 597.

Muir, C. S. and Oakley, W. F. (1966) *J. Lar. Otol.* (In press).

Muir, C. S. and Shanmugaratnam, K.—(1966) In ' Symposium on Cancer of the Nasopharynx and Accessory Sinuses '. Edited by Muir, C. S. and Shanmugaratnam, K. (In Press).

Muir, C. S. and Shanmugaratnam, K.—(1966) In U.I.C.C. Monograph on Cancer Morbidity, edited by Doll, R., Payne, P. M. and Waterhouse, J. A. H. (In Pres.).

Ringertz, N., Törnberg, B., Sjöström, A. and Swenson, D.—(1962). ' Cancer Incidence in Sweden, 1959 '. Stockholm (National Board of Health).

Ringertz, N., Törnberg, B., Sjöström, A. and Swenson, D.—(1963). ' Cancer Incidence in Sweden, 1960 '. Stockholm (National Board of Health).

World Health Organisation—(1957) ' Manual of the International Statistical Classification of Diseases, Injuries, and Causes of Death '. Geneva (World Health Organisation), Vol. 1, p. 45.